# A soft lithographic approach to fabricate InAs nanowire field-effect transistors

**DOI:** 10.1038/s41598-018-21420-y

**Published:** 2018-02-16

**Authors:** Sang Hwa Lee, Sung-Ho Shin, Morten Madsen, Kuniharu Takei, Junghyo Nah, Min Hyung Lee

**Affiliations:** 1 Department of Applied Chemistry, Kyung Hee University, Yongin, Gyeonggi 17104 Korea; 2 Department of Electrical Engineering, Chungnam National University, Daejeon, 34134 Korea; 3 SDU NanoSYD, Mads Clausen Institute University of Southern Denmark, Alsion 2, 6400 Sønderborg, Denmark; 4 Department of Physics and Electronics, Osaka Prefecture University, Sakai, Osaka, 599-8531 Japan

## Abstract

The epitaxial layer transfer process was previously introduced to integrate high-quality and ultrathin III-V compound semiconductor layers on any substrate. However, this technique has limitation for fabrication of sub-micron nanoribbons due to the diffraction limit of photolithography. In order to overcome this limitation and scale down its width to sub-50 nm, we need either a costly short wavelength lithography system or a non-optical patterning method. In this work, high-quality III-V compound semiconductor nanowires were fabricated and integrated onto a Si/SiO_2_ substrate by a soft-lithography top-down approach and an epitaxial layer transfer process, using MBE-grown ultrathin InAs as a source wafer. The width of the InAs nanowires was controlled using solvent-assisted nanoscale embossing (SANE), descumming, and etching processes. By optimizing these processes, NWs with a width less than 50 nm were readily obtained. The InAs NWFETs prepared by our method demonstrate peak electron mobility of ~1600 cm^2^/Vs, indicating negligible material degradation during the SANE process.

## Introduction

The scaling of Si-based metal-oxide-semiconductor field-effect transistors (MOSFETs) has played an important role in achieving high performance devices with low power consumption and has produced tremendous economic benefits^1,2^. This trend has continued to date, following Moore’s law^3^. As the scaling of Si-based devices approaches its fundamental limit, however, alternative channel materials, such as III-V compound semiconductors^4–6^, carbon-based nanomaterials^7–9^, and layered semiconductors^10–12^, have gained attention. Among these materials, III-V compound semiconductors have been considered as next-generation channel materials due to their exceptionally high electron mobility.

Although the performance of III-V MOSFETs exceeds that of Si MOSFETs, high material costs and difficult integration of III-V materials onto conventional Si substrates have hindered the growth of III-V MOSFET industries. Recently, the epitaxial lift-off and transferring (ELT) technique was developed to integrate ultrathin III-V semiconductor layers onto Si/SiO_2_ substrate^13^. This method allows facile integration of different III-V materials with huge lattice mismatches on the same substrate, which has been one of the main obstacles for their use in future device applications. Using this technique, high performance III-V MOSFETs, complementary metal-oxide-semiconductor (CMOS) logic circuits^14^, and radio frequency (RF) circuits^15^ on both Si/SiO_2_ substrates and flexible substrates have been demonstrated. However, this technique has limitation for fabrication of sub-micron nanoribbon-based FETs due to the diffraction limit of photolithography. In order to overcome this limitation and scale down its width to sub-50 nm nanowires (NWs), we need either a costly short wavelength lithography system^16^ or a non-optical patterning method^17^.

Here, we present a modified ELT technique to fabricate sub-50 nm width InAs NWs. Specifically, the InAs NW was fabricated using soft lithography, called solvent-assisted nanoscale embossing (SANE), using epitaxially grown InAs thin film on the buffer layers. Using the transferred NWs on a SiO_2_/Si substrate, InAs NWFETs were fabricated and exhibited high on/off current ratio (~10^4^) and peak electron mobility (~1600 cm^2^V^−1^s^−1^), indicating that our approach is a reliable way to form one-dimensional nanomaterials from 2-D thin films.

## Results

### Fabrication of sub-50 nm InAs NWs using SANE and ELT

**Figure 1:**
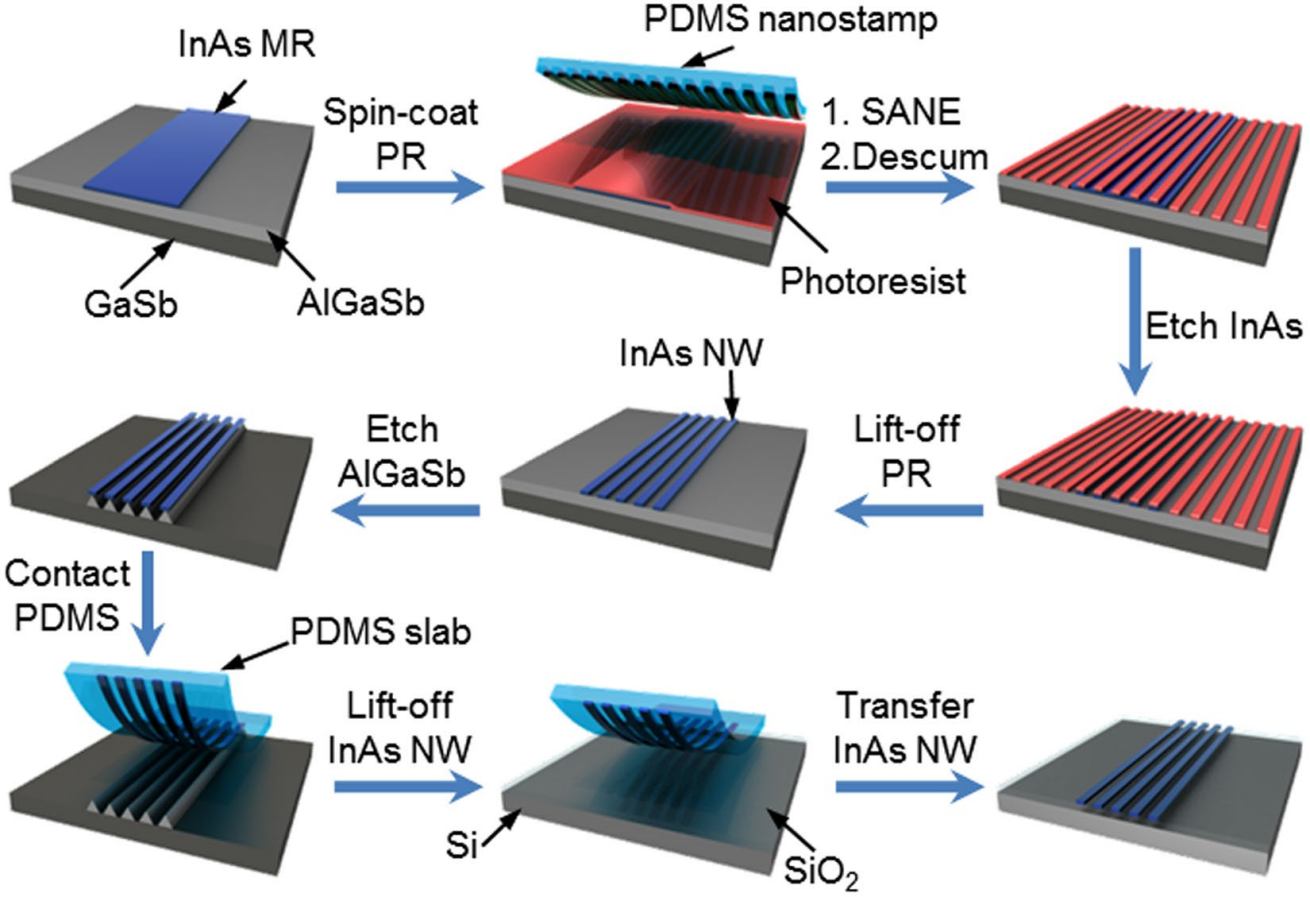
Schematic representation of sub-50 nm InAs NWs transferred to Si/SiO_2_.

Figure 1 shows a schematic representation of fabrication of sub-50 nm NWs using a simple soft lithographic approach. A molecular beam epitaxy (MBE)-grown InAs/Al_0.2_Ga_0.8_Sb/GaSb wafer was used to fabricate the NW. A 5-μm-wide InAs microribbon (MR) was fabricated using conventional photolithography, followed by wet etching of InAs in a mixture of citric acid and H_2_O_2_. The SANE process was then carried out on MRs to create sub-50 nm photoresist (PR) lines on the NRs. A polydimethylsiloxane (PDMS) stamp (with lines 70 nm wide and 140 nm apart), soaked with a drop of dimethyl formamide (DMF), was applied to InAs MRs coated with the photoresist (Shipely S1805). After the DMF was dried, the stamp was detached, leaving uniform PR lines (~50 nm wide and 140 nm apart). Even though residual layers between PR lines were not observable, 5~10 sec of O_2_ plasma descumming was performed to remove potential PR residues. During the descumming process, the width of PR lines was further decreased by increasing the duration of descumming, as described later. Using nano-PR lines as an etching mask, the InAs layer was then etched with a citric acid/H_2_O_2_ mixture, followed by removal of the PR lines. A sacrificial AlGaSb layer was selectively etched using NH_4_OH, forming InAs NWs on an AlGaSb pedestal. A slab of PDMS was then gently pressed against the patterned wafer, which transfers partially released InAs NWs onto the PDMS. The InAs NWs on PDMS were then treated with dilute HF solution (1:50) for 1 min to remove residual AlGaSb. Finally, the PDMS slab with InAs NWs was pressed onto a clean Si/SiO_2_ wafer to transfer the InAs NWs.

### Characterization of sub-50 nm InAs NWs

**Figure 2:**
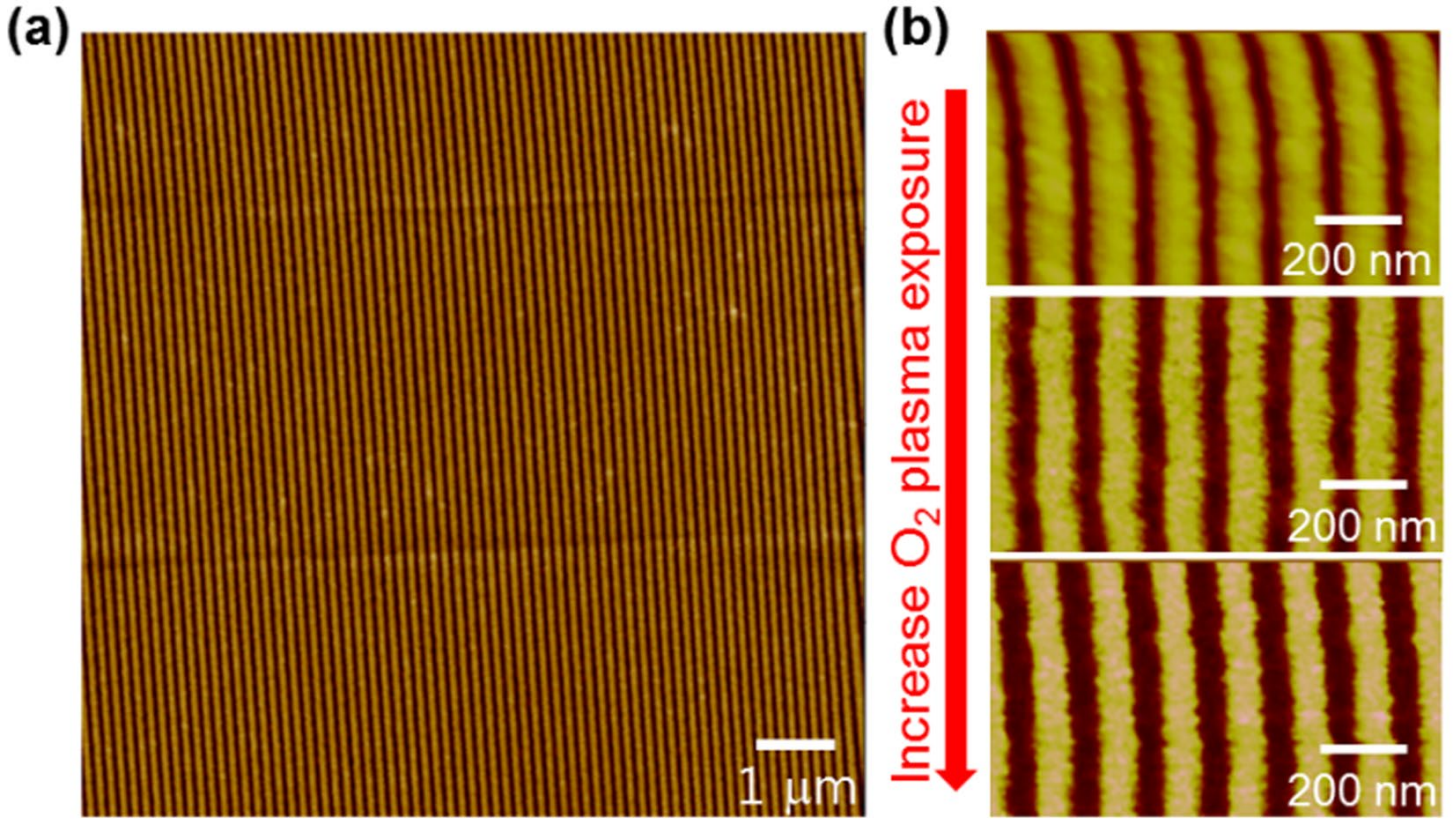
Atomic force micrographs of photoresist line patterns after the SANE process. (**a**) SANE-patterned photoresist over a large area. (**b**) Reduced photoresist line width with increasing O_2_ plasma etching time.

Figure 2a shows the atomic force micrograph (AFM) of uniform PR-line patterns formed over a large area by the SANE process. The SANE process is a critical step in determining the width and electrical properties of InAs NWs for two reasons. First, it can generate sub-100 nm wide PR lines, overcoming the diffraction limit of photolithography. Second, nano-PR lines created by the SANE process are smoother than those created by photolithography. Therefore, the subsequent etching process produces semiconductor NWs with smooth edges, which result in high channel mobility due to reduced scattering from the NW surface^18^. Here, the NW width was reduced by adjusting oxygen plasma processing time on patterned PR lines, where the PR line width was gradually decreased with process time increase (Fig. 2b). However, a long descumming process can cause rough edges on PR lines and reduce the height of PR patterns, necessitating optimization of the process time. After scaling the PR lines, the width of the InAs NWs was further decreased by subsequent InAs wet-etching. We note that the width of PR line patterns can be scaled down further by selecting a solvent with a high swelling factor of the PDMS molds (swelling factor *S* = *D*/*D*_0_, where *D* is the length of PDMS in the solvent, and *D*_0_ is the length of the PDMS in air). As we described in this report, the SANE process using DMF (*S* ~ 1.02) can decrease PR lines by 22% (~50-nm PR line patterns) using the PDMS mold with 70-nm lines. For further width scaling, solvent with a higher *S* such as isopropyl alcohol (*S* ~ 1.09) or dichloromethane (*S* ~ 1.22) can be used, which can reduce the PR lines by 33% (~45 nm PR lines) and 44% (~40 nm PR lines), respectively^19^. Our method is capable of achieving high scaling down factors with all soft lithography methods (SANE and ELT) and producing features down to 30 nm in size without using expensive DUV or e-beam lithography.

**Figure 3:**
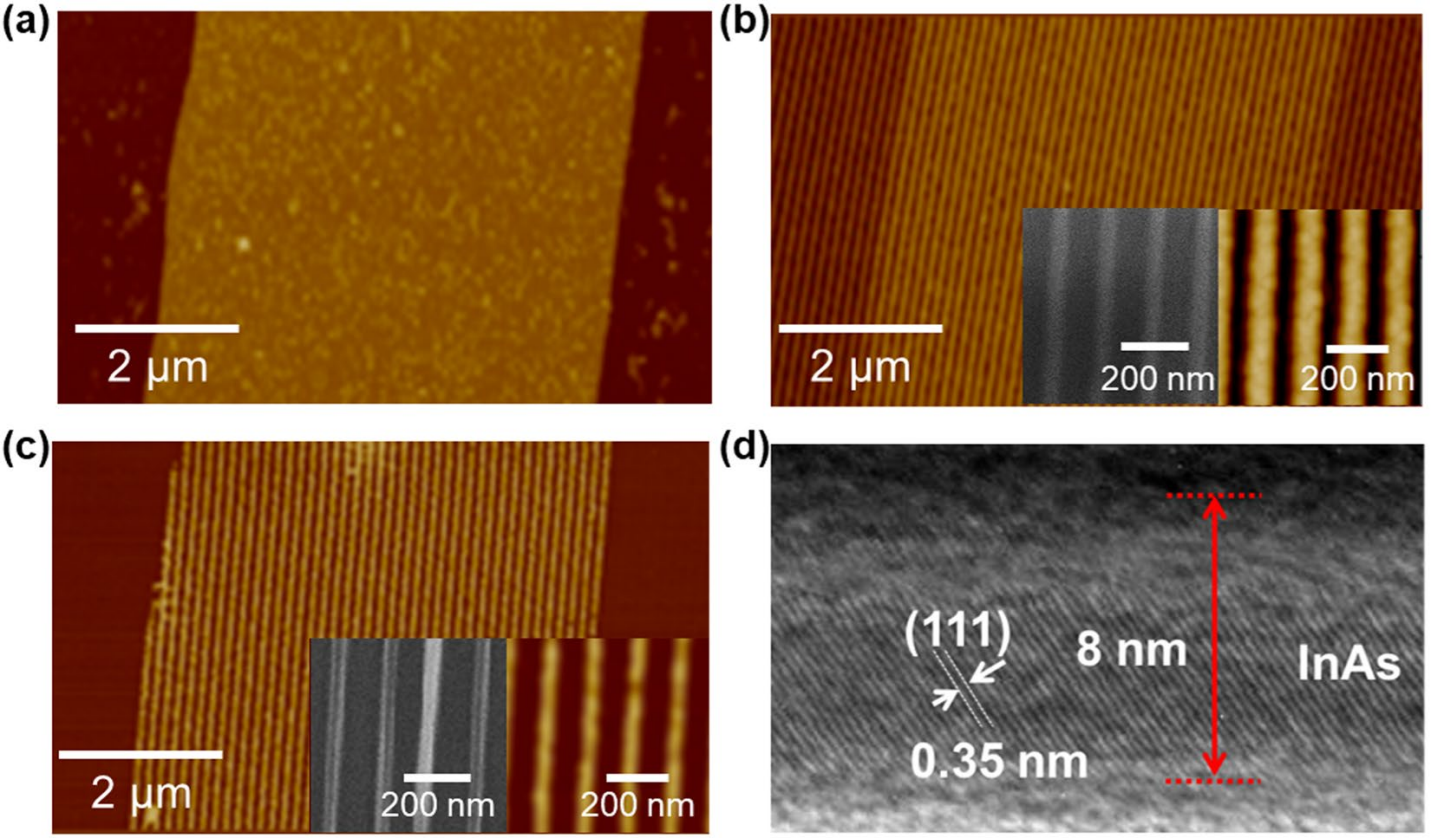
Atomic force micrographs of (**a**) InAs microribbon, (**b**) SANE photoresist pattern on InAs microribbon (inset: SEM and AFM images of 50-nm PR lines), (**c**) InAs NWs (inset: SEM and AFM images of 35-nm InAs NWs), (**d**) Transmission electron micrograph of the InAs NWs transferred onto Si/SiO_2_.

Figure 3 shows AFM images of InAs NWs obtained by SANE and ELT process. The 5-μm InAs MRs were first patterned using conventional photolithography, followed by etching InAs film using micro-PR lines as an etch mask (Fig. 3a). We performed this step to fabricate hierarchical patterns of NWs. After performing SANE on the MR patterned wafers using DMF-soaked PDMS with line patterns (70 nm wide and 140 nm pitch), 50-nm-wide PR lines were created over InAs MRs, exposing the AlGaSb layer (Fig. 3b). The PR lines were decreased by approximately 20% due to swelling of PDMS in DMF^20^. The InAs MRs were then etched again using 50-nm PR lines as an etch mask, producing sub-50 nm InAs NW bundles in each 5-μm section of InAs MR. Afterward, the remaining PR was cleaned with acetone. The InAs NWs transferred onto a fresh Si/SiO_2_ (50 nm) substrate had a width of ~35 nm (Fig. 3c inset) because of undercutting during wet chemical etching. The NW width can be further decreased by increasing InAs etching time. The NW edges and surface were smooth due to fine PR pattern formation after SANE and etching of residual InAs in dilute HF (Fig. 3c). Figure 3d shows a cross-sectional transmission electron micrograph (TEM) of an InAs NW transferred to a Si/SiO_2_ substrate, demonstrating a single crystalline structure. The active InAs layer was ~8 nm thick, and the native oxide was ~2 nm thick.

### Electrical characteristics of the InAs NWFETs

**Figure 4:**
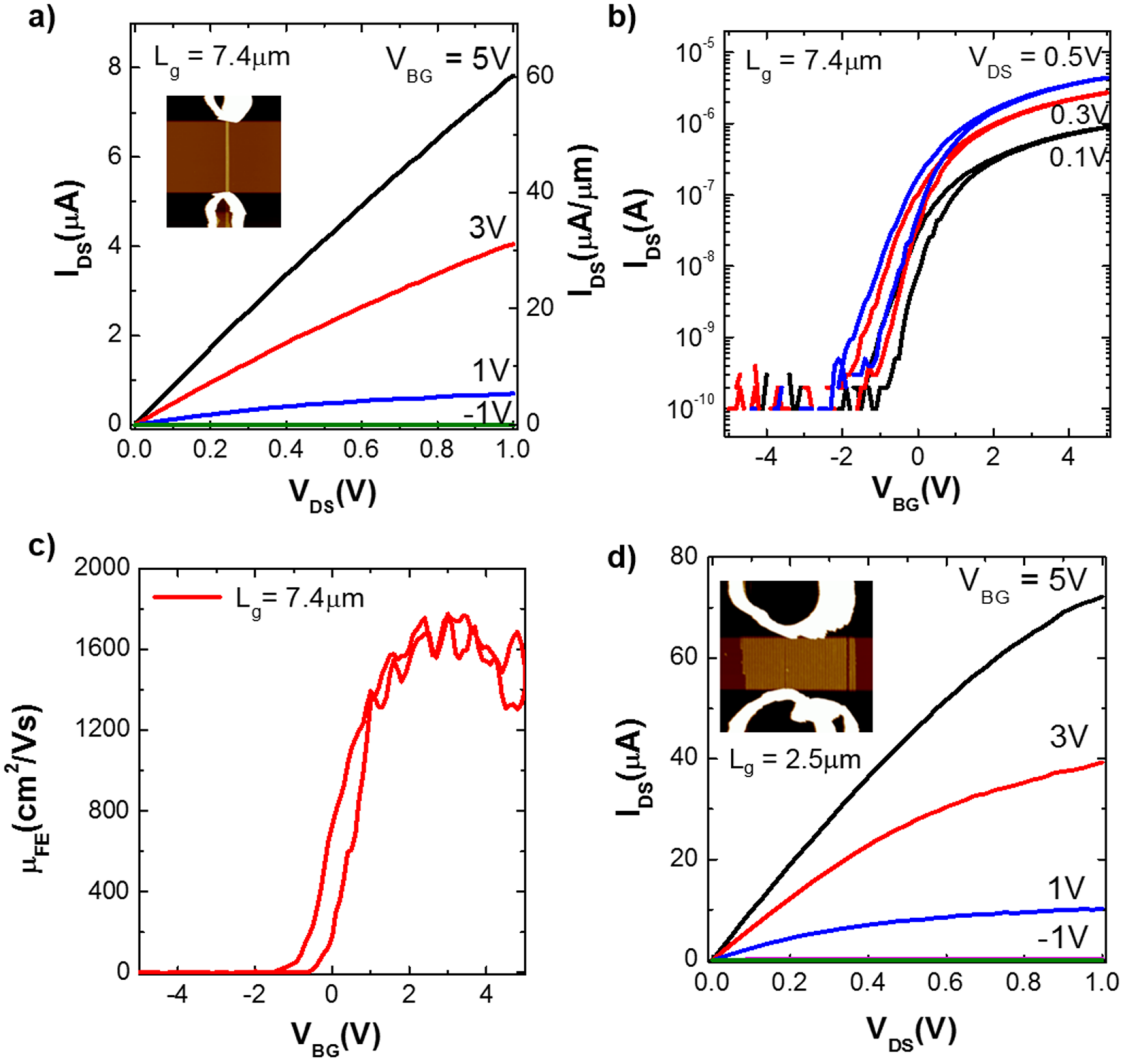
Electrical characteristics of the back-gated InAs NWFETs. (**a**) Output (I_DS_-V_DS_) and (**b**) transfer (I_DS_-V_BG_) characteristics of the InAs NWFET with a channel length of ~7.4 μm and a total NW width of ~130 nm. The right axis in (**a**) shows I_DS_ normalized by the total NW width. (**c**) Field-effect mobility vs. V_BG_, calculated at V_DS_ = 0.1 V for the same device. (**d**) The I_DS_–V_DS_ data of the InAs NWFET with a channel length of high density NW bundles of ~2.5 μm, comprising ~25 NWs.

To evaluate the electrical characteristics of the InAs NWs, we fabricated back-gated NWFETs. The electrical characteristics of the InAs NWFET with three NWs, having a 130 nm total channel width and a 7.4 μm channel length, are shown in Fig. 4a–c. The drain current (I_DS_) increased linearly with V_DS_ until it saturated, suggesting that PR residue from the SANE process does not remain on InAs NWs (Fig. 4a). The InAs NWFET exhibited a high ON-state current of ~33 μA/μm and an OFF-state current below 2 nA/μm at V_DS_ = 0.5 V, resulting in a high ON/OFF current ratio, ~10^4^ (Fig. 4b). The field-effect mobility was calculated as a function of V_BG_ using the standard square law model for electron mobility ${\mu }_{n,FE}={g}_{m}({L}_{g}^{2}/{C}_{ox}{V}_{DS})$, where *g*_*m*_ is the transconductance, ${g}_{m}=d{I}_{DS}/d{V}_{BG}{|}_{{V}_{DS}=0.1V}$ and *C*_*ox*_ is the gate-to-NW capacitance, (Fig. 4c). The calculated peak electron mobility was ~1600 cm^2^/Vs, which excels or is comparable to that of InAs NR FET with the same layer thickness, indicating negligible mobility degradation due to width scaling^13^.

By increasing the number of NWs composing the channel, a NWFET with a channel length of 2.5 μm was fabricated (Fig. 4d) with ~25 InAs NW bundles. The NWFET exhibited approximately 10-fold higher ON-state current by comparison to the data in Fig. 4a, demonstrating that current level modulation can be achieved by adjusting the number of NWs. The number of NWs transferred onto the Si/SiO_2_ substrate can be varied by adjusting the adhesion between the InAs NWs and PDMS stamp. Using *h*-PDMS (hard PDMS) with low adhesion, 3~5 NWs were transferred to the Si/SiO_2_ wafers. With high adhesion PDMS (soft PDMS), high-density NWs can be transferred at a high yield.

## Discussion

In this paper, we report a simple soft nanolithographic approach to obtain NWs from an epitaxially grown III-V wafer. Using this technique, high-quality NWs were reliably produced from a 2 D semiconductor layer, which can be potentially used to realize III-V NWFETs on any substrate. To gauge the quality of NWs, the InAs NWFETs were fabricated and investigated. The enhanced device performance, observed in these NWFETs by comparison to InAs NRFETs^13^, indicates the reliability of our approach. In addition, the device current modulation was demonstrated by controlling the density of transferred NWs. Our approach can also provide a way to create a one-dimensional device structure out of 2 D source materials to study low dimensional physics and can be employed for various device applications.

## Methods

### PDMS nanostamp preparation

hard-PDMS/soft-PDMS nanostamps were prepared by spin-coating hard-PDMS solution onto anti-sticking layer coated Si gratings (70-nm lines, 140-nm pitch, Lightsmyth, USA), followed by coating with soft-PDMS solutions (10:1 ratio mixture of prepolymer and curing agent, Sylgard 184, Dow Corning Co., USA) and curing at 70 °C for 2 hrs.

### InAs microribbon (MR) fabrication

Conventional photolithography was performed on the surface of an i-line PR-coated MBE-grown InAs/Al_0.2_Ga_0.8_Sb/GaSb wafer, using a photomask with microline (5 μm wide and 10 μm pitch) patterns. Using the PR patterns as an etch mask, a 10-nm-thick InAs layer was etched with a mixture of citric acid (1 g per 1 ml of H_2_O) and 30% H_2_O_2_ at 1:20 (v:v), and the PR was removed using ultrasonication with acetone.

### Solvent-assisted nanoscale embossing (SANE)

Photoresist (S1805, Rohm and Hass) was spincoated onto a handling wafer with InAs MRs. The surface of the pre-patterned PDMS nanostamp (lines of 70 nm wide and 140 nm pitch) was soaked with dimethylformamide (DMF) (Sigma-Aldrich) in a crystallizing dish, and it was immediately placed on the S1805-coated InAs MR source wafer. The sample was kept at room temperature without agitation until the DMF was completely dried. Then, the stamp was gently separated from the wafer.

### InAs nanowire (NW) fabrication

The SANE patterned PR lines were treated with oxygen plasma (50 W, 50 sccm) for 5 ~ 10 s to remove residual PR between the nanolines. Using the PR patterns as an etch mask, the InAs MRs were etched using a mixture of citric acid and H_2_O_2_, forming InAs NWs.

### Transfer InAs NWs on Si/SiO2 wafer

To release InAs NWs anchored on the source wafer, the AlGaSb sacrificial layer was anisotropically etched by NH_4_OH (3% in water) solution for 10 min. Next, the 2-mm-thick soft-PDMS slab was gently pressed on the InAs NWs floated on AlGaSb pedestals and was smoothly detached from the source wafer. The InAs NWs on the PDMS slabs were then cleaned in a diluted HF (50:1) solution for 1 min to remove any residues of sacrificial layer on the InAs NWs. The InAs NWs were transferred by gently pressing the PDMS slab onto a Si/SiO_2_ (50-nm thick thermally grown SiO_2_) wafer.

### NWFET fabrication

The back-gated NWFETs were fabricated by depositing Ni (50 nm) on photolithographically defined source/drain regions of the transferred InAs NWs.
